# Dark-Field Scattering and Local SERS Mapping from Plasmonic Aluminum Bowtie Antenna Array

**DOI:** 10.3390/mi10070468

**Published:** 2019-07-13

**Authors:** Thang Duy Dao, Chung Vu Hoang, Natsuki Nishio, Naoki Yamamoto, Akihiko Ohi, Toshihide Nabatame, Masakazu Aono, Tadaaki Nagao

**Affiliations:** 1 International Center for Materials Nanoarchitectonics (MANA), National Institute for Materials Science (NIMS), 1-1 Namiki, Tsukuba 305-0044, Japan; 2 Graduate School of Materials Science, Nara Institute of Science and Technology, 8916-5 Takayama, Ikoma, Nara 630-0192, Japan; 3 Institute of Materials Science (IMS), Vietnam Academy of Science and Technology (VAST), 18 Hoang Quoc Viet street, Hanoi 100000, Vietnam; 4 Institute of Theoretical and Applied Research (ITAR), Duy Tan University, 1 Phung Chi Kien Street, Hanoi 100000, Vietnam; 5 Physics Department, Tokyo Institute of Technology, Meguro-ku, Tokyo 152-8551, Japan; 6 Department of Condensed Matter Physics, Graduate School of Science, Hokkaido University, Kita-10 Nishi-8 Kita-ku, Sapporo 060-0810, Japan

**Keywords:** aluminum, plasmonics, dark-field scattering, SERS, bow-tie antenna

## Abstract

On the search for the practical plasmonic materials beyond noble metals, aluminum has been emerging as a favorable candidate as it is abundant and offers the possibility of tailoring the plasmonic resonance spanning from ultra-violet to the infrared range. In this letter, in combination with the numerical electromagnetic simulations, we experimentally study the dark-field scattering spectral mapping of plasmonic resonance from the free-standing Al bowtie antenna arrays and correlate their strong nearfield enhancement with the sensing capability by means of surface-enhanced Raman spectroscopy. The spatial matching of plasmonic and Raman mapping puts another step to realize a very promising application of free-standing Al bowtie antennas for plasmonic sensing.

## 1. Introduction

Alternative plasmonic materials beyond gold (Au) and silver (Ag), including transition metal nitrides and metal carbides [1,2,3,4], heavily-doped semiconductors [5,6,7,8,9], as well as graphene [10,11,12,13,14,15], have been widely investigated recently owing to their controllability of optical properties via chemical or physical doping processes. Being the third most abundant element on earth, after silicon and oxygen, aluminum (Al) has also been used widely in industry. In the past few years, aluminum have attracted tremendous interest in the field of plasmonics as it is a promising alternative replacement for noble plasmonic metals [16,17,18,19,20]. Having an ultrathin native oxide Al_2_O_3_ layer (2 nm–4 nm), Al emerges as a favorable material in the field of plasmonic sensing with the detection mechanism mainly based on the electromagnetic field enhancement rather than the charge transfer effect [16,17,21,22]. Compared to Au and Ag whose interband transitions are at ~2.3 eV and ~3.9 eV [23], respectively; Al has a lower interband transition energy of about ~1.5 eV. Aluminum displays a plasma energy of 15.3 eV thatis higher than that of Au and Ag, and, its energy loss function Im(−1ε), wherein *ε* is the dielectric function of the material, offers no maxima of dielectric loss in the range from UV to VIS, thus making Al an excellent Drude metal in this range [24,25,26,27]. In terms of the optical absorption, the absorption efficiency of Al nanoparticles is greater than that of Au and Ag, and is slightly smaller than that of alkali metals [28]. Therefore, plasmonic resonance of Al nanoantennas can be flexibly tuned over the UV-VIS to the IR region, just by adopting suitable nanostructured architectures [29,30,31,32,33,34].

In the past decades, plasmon-enhanced vibrational spectroscopic sensing including surface-enhanced Raman scattering (SERS) [35,36] and surface-enhanced infrared absorption spectroscopy (SEIRA) [37,38,39] have showed great advantage over the conventional spectroscopic methods in the trace molecular detection, especially for monolayer and single molecule via specific surface chemical functionalization [40,41,42,43,44,45]. In the field of SERS, many plasmonic antennas have been proposed for SERS substrates including plasmonic nanospheres [40,46], nanorods [47,48], nanotriangles [17,49], particularly the structures having narrow gaps such as aggregate nanoparticles [50], nanoparticle dimers [46,51], nanoclusters [51,52], and nano-bowties [22,53,54]. Among them, plasmonic nano-bowties have attracted much attention because of its largest nearfield enhancement that could be achieved at the nanogaps between the two triangles [22,53]. On the other hand, the Raman cross section is increased in the short wavelength(*λ*) region (UV-NIR) as a function of *λ*^−4^, however, engineering the resonance of bowtie antennas in the UV-NIR region using Au and Ag is impossible due to their small plasma frequencies and the fabrication limit. Therefore, utilizing plasmonic Al for nanobowtie antennas can realize short-wavelength resonant antennas (500 nm–700 nm) for SERS applications.

In this letter, we numerically and experimentally demonstrated free-standing Al bowtie antennas for SERS study. As the group velocity of the “slow light” formed by the coupling of plasmon and light is screened by the dielectric property of the surrounding media, thus, having an equality of the group velocity by a homogeneous surrounded dielectric medium between the upper and lower metal/dielectric interfaces would give a better homogeneity of the distribution of the electromagnetic field [54]. Compared to the previous Al bowtie antennas for SERS that the Al bowties was placed directly on a fused silica substrate [22], in this work, by introducing a SiO_2_ post under each Al triangle, the nanogap area becomes free from the dielectric screening and thus the induced field enhancement at the nanogaps located between two plasmonic Al triangles could be increased and occupy a larger available volume for accommodating analyte molecules. We investigated the dark-field scattering mapping spectra of the plasmonic free-standing Al bowtie antennas, for correlating the obtained resonance with their sensing capability by the surface-enhanced Raman spectroscopy (SERS) of organic molecules. Supported by the numerical simulations, we showed that the free-standing Al bowtie arrays could be a powerful antenna platform for SERS. Our result also paves a way for the possible applications of the oxide-coated Al bowtie array serving as strong near-field optical antennas for SERS as well as other plasmon-enhanced spectroscopic devices.

## 2. Materials and Methods

**Figure 1:**
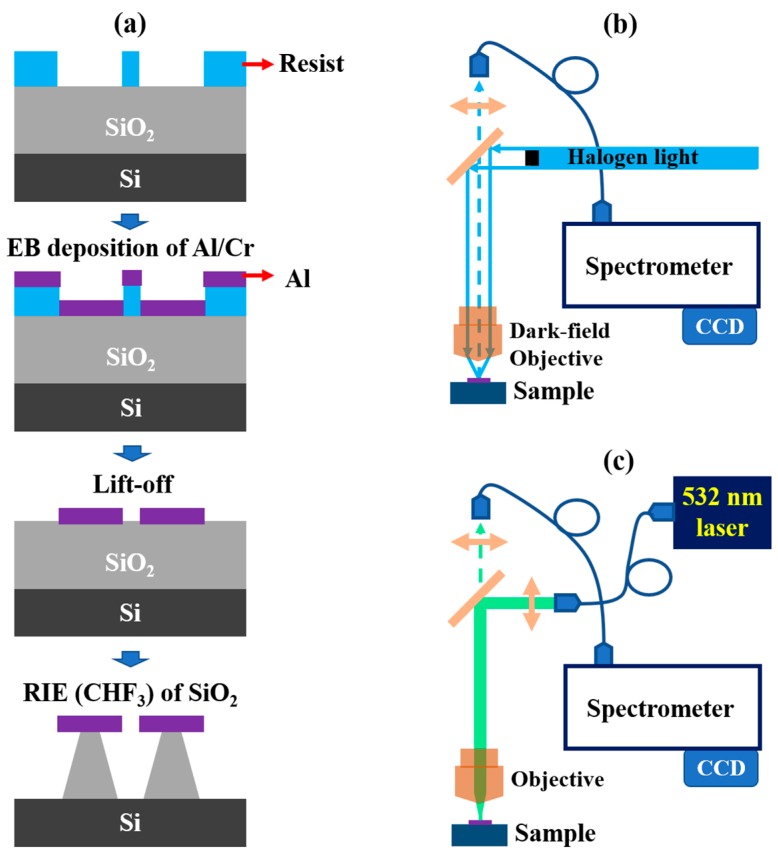
(**a**) Schematic illustration of the fabrication process. Optical microscopy setup of (**b**) dark-field measurement and (**c**) Raman measurement.

The free-standing Al bowtie nanoantennas were fabricated by a standard procedure using electron beam (EB) lithography, lift-off, and reactive-ion etching (RIE) processes (Figure 1a). Prior to the fabrication, a 200-nm-thick layer of SiO_2_ was prepared by the thermal oxidation of a 1 × 1 cm^2^ Si wafer. An EB resist mask was prepared on a 200-nm-SiO_2_/Si substrate using an EB writer (Elionix, ESL-7500DEX, Tokyo, Japan). A 40-nmthick of Al with a 4-nm-adhesive Cr layer was then deposited on the substrate with the EB resist mask using EB deposition (UEP-300-2C, ULVAC, Kanagawa, Japan). After the lift-off step, an RIE process (ULVAC CE-300I, Kanagawa, Japan) with a mixture of CHF_3_/O_2_ (15-sccm/2.5-sccm) gases was applied to etch a 180-nmdepth SiO_2_ layer with Al bowties as mask, forming a 180-nm height SiO_2_ post underneath each Al triangle. The morphology of the fabricated Al bowtie arrays was characterized by using a field-emission scanning electron microscope (FE-SEM, Hitachi SU8000, Tokyo, Japan).

A con-focal Raman microscope (WITec Alpha 300S, Ulm, Germany) combined with a halogen lamp (Lucir-LAHL100) in UV-NIR range and a second harmonic diode pumped Nd:YAG laser (WITec, Ulm, Germany) at 532 nm was used for the investigation of the plasmonic scattering property (dark-field geometry, see Figure 1b) and the demonstration of the SERS effect (Figure 1c). As for the dark-field scattering measurements, a scanning mode equipped with a 50× dark-field lens (Zeiss, Oberkochen, Germany) was used. The relative scattering spectra were obtained by subtracting the spectra taken from the free-standing Al bowtie antennas with posts supported by the Si substrate to the spectra taken from a bare Si sample.

To demonstrate the SERS effect, a 10 µL droplet of Nile-blue solution (concentration 3 µM) was spread over the Al bowtie arrays (1 × 1 cm^2^ substrate) and then dried in ambient condition. The SERS mapping measurements were performed on an area of 10 µm × 10 µm, with a spatial resolution of 40 pixel (250 nm/pixel), an integration time of 0.2 s/pixel. A 100× objective lens was used with an optimal laser power of 500 µW.

The rigorous coupled-wave analysis (RCWA) (DiffractMOD, Synopsys’ RSoft, Version 2017.09, Ossining, NY, USA) and the finite-difference time-domain (FDTD) methods (FullWAVE, Synopsys’RSoft, Version 2017.09, Ossining, NY, USA) were employed to get further understanding on the optical properties of the plasmonic Al bowties as well as the enhancement nature of the electromagnetic field at their gaps and surfaces. The dielectric functions of Al, Cr, Si and SiO_2_ were referred from the literature [55,56]. The model of the free-standing Al bowtie structures used for the simulations was constructed based on a build-in CAD layout (RSoft CAD Environment™, Version 2017.09, Ossining, NY, USA). Each free-standing bowtie antenna composed of a Si substrate and two SiO_2_ posts (height of 180 nm) and Al bowties. The Al bowties have the triangle shapes (length of 105 nm, thickness of 40 nm), and composed of a 4-nmthick native Al_2_O_3_ oxide layer on top and 4-nm-thick Cr adhesive layer underneath. To mimic the real structures, the triangular truncations were placed at the corners of the triangles. The periodic boundary and grid size conditions were optimized to simulate the whole structure.

## 3. Results

**Figure 2:**
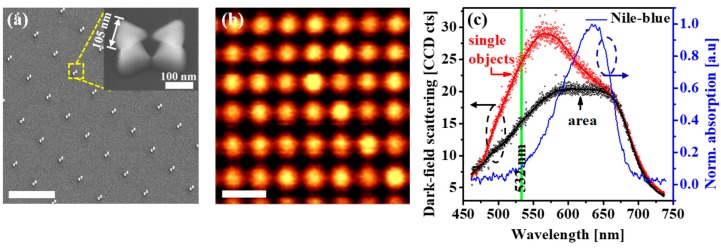
Spectral mapping of the free-standing Al bowtie antennas. (**a**) A scanning electron microscope (SEM) of the Al antenna arrays, observed at 30° oblique angle and the inset of one typical bowtie antenna. The antennas are spatially distributed with periodicity of 1400 nm, spacing of 1120 nm, 105 nm length and 20 nm gap. (**b**) The spectral mapping integrated over the resonance of the antennas, ranging from 500 nm to 700 nm. The circular “hot-spots” are spatially distributed following the SEM image, as shown in (**a**). The scale bar is 2 µm for both (**a**) and (**b**). (**c**) Spectral response of the single antenna (red), antenna array (black) and absorption spectrum of Nile-blue (blue), details in the text.

Figure 2a presents the tilted-view FE-SEM image of an Al bowtie antenna array with parameters of 105 nm length, 20 nm gap, 1400 nm periodicity (the distance between two bowties), and 1120 nm spacing (the distance between two bowtie lines). The inset of Figure 2a shows a bowtie antenna standing freely on the SiO_2_ posts. The spectroscopic measurements of the Al bowtie antennas are presented in Figure 2b,c. As the obtained spectra are achieved by subtracting the dark-field scattering spectrum taken from the area with the presence of bowtie antennas with respect to the reference area of the bare Si sample, it is hence inferred that our measurement reflects the plasmonic characteristics in the far-field of the antenna resonances. Under the near-field measurement, in the particular case of bowtie antennas, the resonance spectrum depends on the excited position of each single object. For example, the bowtie gap-mode (bright-mode) and dark-mode are seen under the excitation at the point near the gap, and at the point located far from the gap, respectively [57]. In the far-field measurement, the resonance spectrum is a superimposition of the scattering, emission, and interaction from the objects. There exists a difference between the near-field and far-field resonance. The far-field measurement reflects the resonant nature (the polarization is perpendicular or parallel to the bowtie axis) of the plasmonic nanoantennas and does not influence much on the SERS effect. In contrast, the near-field resonance does affect the SERS signals [58]. As seen in Figure 2b, the dark-field scattering spectral mapping of plasmonic resonance from the Al bowtie antenna array (taken in a range from 500 nm–700 nm of the resonance peak) indicates the far-field resonance of the antenna array, which reveals a clear spatial distribution of the circular spots following the periodicity of the actual antenna array.

A comparison of the spectral characteristic between the single antenna and a scanned antenna array is shown in Figure 2c, together with the absorption spectrum of the organic Nile-blue solution, which was intentionally used as the probe molecules to demonstrate the field enhancement thanks to the SERS effect. The normalized dark-field scattering spectrum of the single objects shows a prominent plasmonic peak located at around ~565 nm and a noticeable hump at ~675 nm, toward the longer wavelength region. In the spectrum averaged from an antenna array consisting of the individual objects, the interaction between them and the surrounding areas, it is obvious that the low energy (long wavelength) peak still remained, but the main resonance peak is no more dominant.

**Figure 3:**
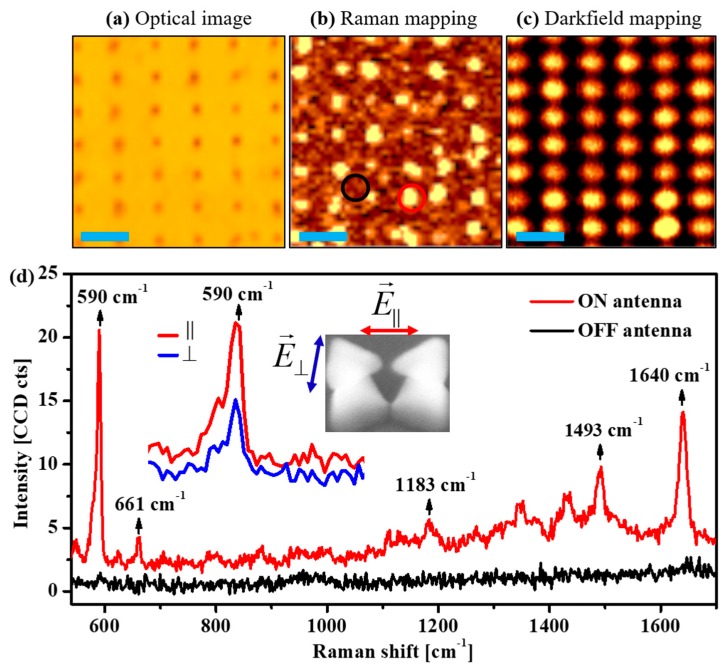
Demonstration of the electromagnetic field enhancement of free-standing bowtie antennas by SERS. (**a**) Optical image (seen under confocal microscope), (**b**) Raman mapping, and (**c**) corresponding plasmonic dark-field scattering mapping of the antenna arrays, respectively. The scale bar indicates 2 µm in all (**a**), (**b**), and (**c**). (**d**) SERS measurements of the Nile-blue molecules on the Al bowtie antennas. The black curve and red curve show Raman signals “OFF” and “ON” the antennas. The small inset indicates the Raman signals under different excitation conditions; with polarization parallel to the bowtie axis (red) and perpendicular to the bowtie axis (black), respectively.

Figure 3 shows the SERS measurements of the free-standing Al bowtie antennas with Nile-blue molecules. Very different from Au and Ag surfaces, which allow for achieving a well-defined chemical adsorption of a single layer of probe molecules (octadecanethiol (ODT), for instance), giving a precise estimation of the electromagnetic enhancement factor of the plasmonic nanoantennas [39]; the Al antennas are naturally coated by a few nanometer-thick Al_2_O_3_ that protects the Al surface from directly contacting the probe molecules. However, the Al_2_O_3_ is a fairly inert and reusable surface making it a promising SERS template for practical applications. The use of the Al_2_O_3_/Al configuration for the SERS measurements can solely demonstrate the electromagnetic field enhancement effect on SERS without any influence of the charge transfer (also known as chemical effect) between the plasmonic antennas and the Nile-blue molecules. The SERS performance of the free-standing Al bowtie arrays was investigated by evaluating the enhancement of the Raman spectrum of Nile-blue molecules. Figure 3a–c show an optical image (seen by confocal microscope), a reconstructed Raman mapping image (integrated at 590 cm^−1^ band), and a dark-field scattering mapping image of the corresponding area, respectively. Figure 3d presents the SERS spectra of Nile-blue molecules at the position “ON” the Al bowtie (red spectrum) and at the position “OFF” the Al bowtie (black spectrum). It is obvious that the Raman signals of Nile-blue fingerprints are strongly enhanced if the molecules are located at the position of Al bowties, giving clear evidence of the promising SERS effect due to the strong nearfield enhancement at the small gaps of the free-standing Al bowtie antennas. Additionally, the measurement of the polarization-dependent SERS was performed by using a half-wave plate to change the polarization of the excitation laser. The inset in Figure 3d shows that the relative intensity of Raman signals at the 590 cm^−1^ peak in the case of the excitation laser with polarization parallel to the bowtie axis (red curve) is significantly higher than that of the perpendicular polarization excitation (black curve). As the Nile-blue molecules cover the bowtie antennas entirely, the overwhelming Raman signal of parallel polarization compared to the perpendicular polarization excitation reflects that the gap-mode (excited under parallel polarization) gives a larger enhancement than the quadrupolar mode (excited under perpendicular polarization), which is in agreement with the previous report [59].

**Figure 4:**
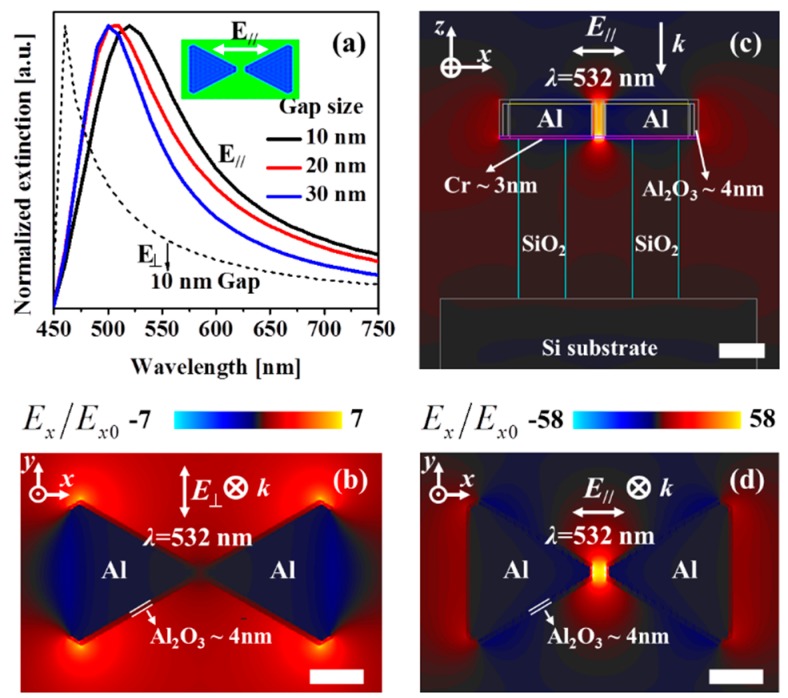
Simulated optical response and electric field distributions around Al bowtie excited at 532 nm wavelength. (**a**) Extinction spectra of the Al bowtie antennas at two different polarization conditions: parallel to the bowtie axis with different gap size of 10 nm, 20 nm, 30 nm (solid curves), and perpendicular to the bowtie axis with a 10 nm gap (dashed curve). The electric field distributions around a 105-nm-length, 10-nm-gap Al bowtie array excited at the 532-nm wavelength are simulated with two polarized excitations: (**b**) Perpendicular polarization, (**c**) and (**d**) parallel polarization with side-view and top-view, respectively. The scale bar is 50 nm for all panels (b), (c) and (d). The electric field hot-spot shows a strong electric field confinement with an enhancement factor |*E*_x_/*E*_x0_| of 58, where *E*_x_ and *E*_x0_ are the amplitudes of the enhanced and incident electric fields, respectively.

The plasmonic resonance of the Al free-standing bowtie antennas was further elucidated using the numerical electromagnetic simulations; details are described in the Materials and Methods Section. Figure 4a shows the simulated extinction spectra of the floating Al bowtie antennas under two incident electric field polarizations; parallel to the bowtie axis with different gap sizes of 10 nm, 20 nm, 30 nm (solid curves), and perpendicular to the bowtie axis with agap of 10nm (dashed curve), separately. When the incident electric field oscillates parallel to the bowtie axis, the extinction spectrum for a 10-nm-gap antenna array shows a clear single resonance at 540 nm, and it is blue-shifted when the gap size increases. On the other hand, when the incident electric field oscillates perpendicular to the bowtie axis, the extinction spectrum of a 10-nm-gap bowtie antenna array tends to shift the resonance to the shorter wavelength region (see the dashed curve in Figure 4a). In comparison to the extinction spectrum measured on a single bowtie presented in Figure 2c, the simulated spectrum indicates a single peak, located closely to the main measured peak (560 nm). However, the simulation could not retrieve the second—(hump-like) peak at 670-nm. This discrepancy could be assigned to the fact that the free-standing architecture of the bowtie antennas results in a relatively strong far-field interaction, which could be seen only by far-field scattering experiments, not in the nearfield simulations [58]. Figure 4b shows the electric field distribution of Al bowtie antennas under the excitation of 532nm with the incident electric field polarized perpendicular to the bowtie axis. In this case, the excited hot-spots are located at the corners of each triangles and the field enhancement was found to be about *E*_x_/*E*_x0_ = 7, where *E*_x_ and *E*_x0_ are the amplitudes of the enhanced and incident electric fields, respectively. Figure 4c,d show the electric field distribution of the Al bowtie antennas array with the incident electric field polarized parallel to the bowtie axis, under the same wavelength excitation of 532 nm; side-view (Figure 4c) and top-view (Figure 4d). It has been previously shown that the electric field enhancement decreases as the gap size of the bowtie increases [53,54]. As shown in Figure 4c,d, the electric field is mainly concentrated at the nanogap of the bowtie antennas. The enhanced area is homogeneously distributed over the gap and the enhancement factor (*E*_f_) is calculated to be about |*E*_x_/*E*_x0_| = 58, which is much large rcompared to that of the perpendicular polarization excitation. It is consistent with the SERS results as discussed above where the SERS signal intensity under the electric field polarized parallel to the bowtie axis is stronger than that of the electric field polarized perpendicular to the bowtie axis (Figure 4d).

## 4. Conclusions

In summary, we have numerically and experimentally demonstrated short-wavelength plasmonic Al bowtie antennas for SERS application at the 532-nm excitation. The free-standing Al bowtie antennas exhibited a strong electric field enhancement as high as 58 when excited at the 532 nm. We also further investigated optical properties of the fabricated free-standing Al bowtie antennas by introducing a plasmonic dark-field scattering mapping, which revealed the resonance of each individual antenna in the range between 500 nm–675 nm. Despite of having a natural oxide Al_2_O_3_ layer, and the absence of the chemical SERS effect, the correlated SERS mapping of Nile-blue molecules on Al bowties excited by a 532-nm laser clearly showed that the SERS signals were observed only at the bowtie antenna position. This evidences that the free-standing Al bowties exhibited a high SERS performance owing to the strongly confined electromagnetic field at the nanogaps between each triangle-pair of the bowtie antennas. Together with a recently proposed surface functionalization method of Al nanoantennas by using phosphoric acid [34], this work puts another step towards the practical applications of aluminum nano antennas in plasmonic sensing as well as in plasmon-enhanced nanospectroscopy.
